# The gut and heart’s role in reward processing

**DOI:** 10.3389/fnint.2025.1479923

**Published:** 2025-07-02

**Authors:** Minel Arinel, Karim Abdelaal

**Affiliations:** ^1^Naumann Laboratory, Department of Neurobiology, Duke University School of Medicine, Durham, NC, United States; ^2^The Collective for Psychiatric Neuroengineering, Department of Neurobiology, Duke University School of Medicine, Durham, NC, United States

**Keywords:** reward, gut-brain, heart-brain, adaptive behavior, bioenergetics, interoceptive therapeutics

## Abstract

Reward processing, which ensures survival, has evolved to also shape emotions, learning, and overall well-being. While traditional models of reward have focused predominantly on central neural circuits, emerging evidence underscores the role of peripheral bodily signals. This represents a new opportunity by which we may understand neurological and neuropsychiatric health. In this review, we explore the gut-brain and heart-brain interfaces in reward processing, delineating their contributions across distinct phases of reward and offering insights into their bioenergetic significance. By framing this interplay within an adaptive and clinical context, we propose new avenues for understanding and treating neuropsychiatric disorders through a mind–body medicine lens.

## 1 Introduction

**Figure 1:**
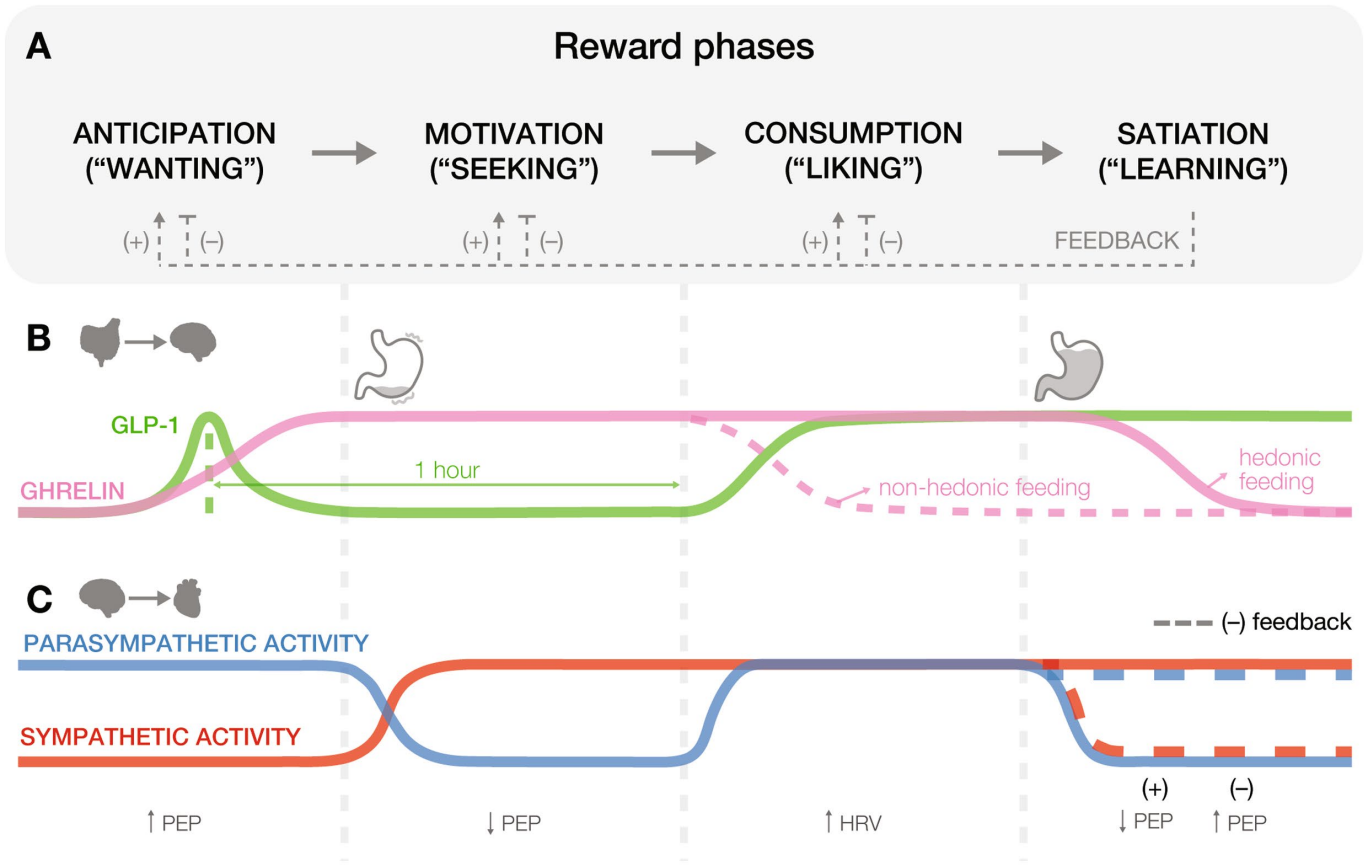
Gut and heart physiology across reward processing phases. **(A)** Reward processing unfolds in four phases: anticipation (“wanting”), motivation (“seeking”), consumption (“liking”), and satiation (“learning”). Positive (+) and negative (−) feedback loops emerge during the satiation phase, influencing how earlier phases are re-engaged in future cycles. Positive feedback, resulting from pleasurable stimuli, reinforces learning and increases future anticipatory, motivational, and consummatory responses toward similar rewards. In contrast, negative feedback, arising from aversive or unsatisfying stimuli, dampens the reward cycle by decreasing anticipatory, motivational, and consummatory responses to those stimuli. These feedback mechanisms function as internal updates to optimize future reward-seeking behavior based on past experiences. **(B)** Gut peptides influence hedonic eating and food preferences at various stages of feeding and reward. Hormones like ghrelin and glucagon-like peptide-1 (GLP-1) exhibit dynamic changes that drive food-related behaviors, balancing hunger and satiety cues. **(C)** Cardiac physiology responds to reward states through parasympathetic and sympathetic activity, as reflected in biomarkers like heart rate variability (HRV) and pre-ejection period (PEP).

Reward processing systems have developed to regulate internal states, promote well-being, and motivate adaptive behaviors essential for survival and reproduction (O’Connell and Hofmann, 2011). These systems, which are shaped by evolutionary pressures, organize adaptive decision-making into a series of phases that include anticipation, motivation, consumption, and a post-consummatory learning phase often referred to as satiation (Figure 1A). For example, as temperatures drop in late autumn, a mouse’s diminishing energy stores trigger hunger (anticipation) and prompt a search for food in familiar foraging sites or caches (motivation). Eating the food reward (consumption) elicits hedonic pleasure, facilitating memory consolidation (satiation) of the locations, conditions, and food availability that reinforce future behavior and enhance survival. Throughout this process, the gut communicates with the brain to coordinate internal states with behavior, first by releasing hunger signals that prompt food seeking, then by gradually shifting to satiety signals as nutrients are detected. In parallel, the heart rate decelerates in anticipation of reward, rises during energy-demanding seeking, and stabilizes following consumption (Graham and Clifton, 1966; Eubanks et al., 2002). This seamless coordination between internal systems and the brain across reward processing phases is essential for adaptive behavior and survival. However, clinical populations often exhibit dysfunctions in these stages of reward processing, manifesting as misjudgments in the value, desirability, or predictability of pleasurable outcomes (Linnet, 2014; Volkow and Morales, 2015; Serretti, 2023). And while traditional frameworks of reward processing in healthy and disordered states have predominantly focused on the central nervous mechanisms, there is much to learn about the role of bodily signals in regulating these processes.

Interoception is a mechanism evolved to support homeostasis and adaptive behavior by synergizing bodily signals (Chen et al., 2021). Given the extensive research on the roles of the gut and heart in mediating interoception, we will focus this review on these organs. The gut and heart are both essential for linking survival needs with adaptive behaviors as they each provide critical, real-time feedback to the brain about the body’s internal states (Chen et al., 2021). These organs can also directly contribute to homeostasis and reward processing, shaping an organism’s capacity to anticipate, engage with, and learn from rewarding stimuli. Governing energetic demands, visceral sensations, and autonomic control, the gut and heart work in concert with the brain to fine-tune reward processes and organize adaptive, energy-efficient decisions that optimize survival and well-being (Kimura, 2019; Liu and Bohórquez, 2022). Emerging research highlights the interplay between these peripheral systems and central reward circuits, offering new avenues to understand and treat neuropsychiatric disorders (Zheng et al., 2009; Critchley and Harrison, 2013; Seth, 2013; Karaivazoglou et al., 2024). This mini-review explores the role of gut-brain and heart-brain communication in reward processing, considering perspectives that underscore the adaptive significance of these interactions.

## 2 Gut-brain and heart-brain signals in reward processing

### 2.1 Gut-derived signals in reward processing

**Figure 2:**
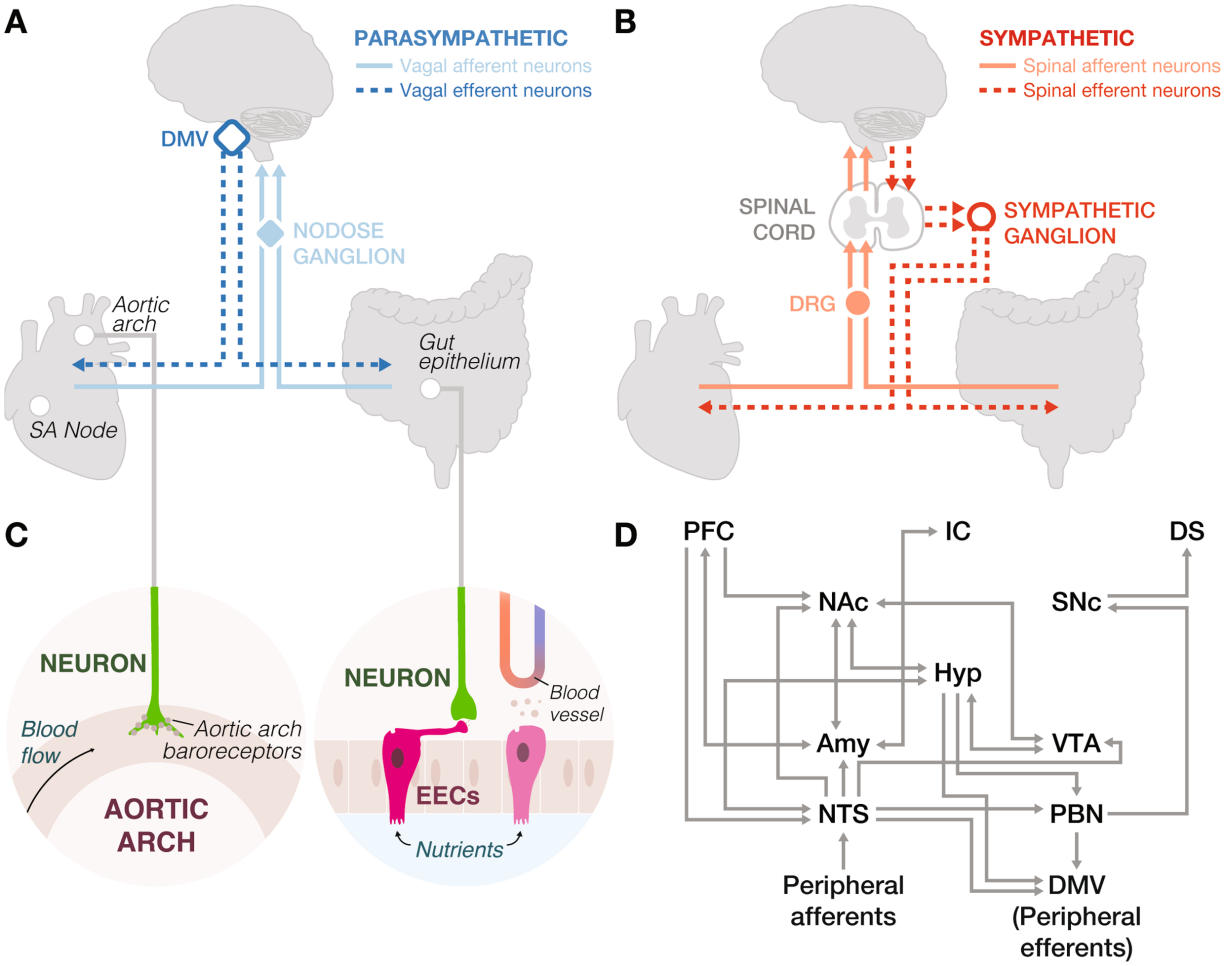
Peripheral innervation and neural circuits underlying interoception. **(A)** Parasympathetic nervous system: Vagal afferent neurons in the nodose ganglia transmit sensory signals from the sinoatrial (SA) node and gut epithelium to the brainstem. These signals are processed and relayed back to the body via vagal efferent neurons in the dorsal motor nucleus of the vagus (DMV). **(B)** Sympathetic nervous system: Spinal afferent neurons in the dorsal root ganglia (DRG) relay sensory information from the periphery to the spinal cord, which is then transmitted to the brain. Spinal efferent neurons project from the spinal cord to sympathetic ganglia, innervating peripheral organs. **(C)** Left: Peripheral neurons in the aortic arch express baroreceptors to detect changes in arterial pressure. Right: Enteroendocrine cells (EECs) sense nutrients, mechanical stretch, and microbial metabolites, and secrete hormones into the bloodstream. However, they can also form synapses with peripheral neurons to send fast signals to the brain. **(D)** Central integration of gut and heart signals involves the central autonomic network and dopaminergic systems, connecting to higher-order brain regions. This circuitry underscores the complex interplay between interoceptive inputs and reward processing. Amy: amygdala, DMV: dorsal motor nucleus of the vagus, DS: dorsal striatum, Hyp: hypothalamus, IC: insular cortex, NAc: nucleus accumbens, NTS: nucleus tractus solitarius, PBN: parabrachial nucleus, PFC: prefrontal cortex, SNc: substantia nigra pars compacta, VTA: ventral tegmental area.

The gut senses diverse internal stimuli, such as nutrients, distension, and microbial metabolites, and influences reward processing through both hormonal and neural pathways. These influences fall into three domains: hormonal signaling, synaptic signaling, and microbiota (not covered here, see: González-Arancibia et al., 2019; García-Cabrerizo et al., 2021; de Wouters d’Oplinter et al., 2022). Gut epithelial enteroendocrine cells (EECs) detect these signals and communicate with the brain through multiple mechanisms (Gribble and Reimann, 2016), including slow systemic release of hormones, or rapid synaptic communication with vagal and spinal neurons through a subset of EECs known as neuropod cells (Bohórquez et al., 2015; Bellono et al., 2017; Kaelberer et al., 2018; Figures 2A–C). Together, these pathways enable the gut to modulate reward circuits across varying timescales.

Ghrelin and glucagon-like peptide-1 (GLP-1) are two key gut-derived peptides that influence reward. Ghrelin primes the stomach for food intake by stimulating gastric acid secretion and motility via vagal pathways (Masuda et al., 2000), aiding digestion and generating interoceptive signals perceived as hunger (Carlson, 1993). In rats and humans, elevated ghrelin levels track hedonic (Merkestein et al., 2012; Monteleone et al., 2012; Rigamonti et al., 2015) and caloric (Hogenkamp et al., 2013) values of an anticipated meal, enhancing food motivation (Figures 1A,B: ANTICIPATION). Interestingly, blocking GLP-1 receptors (GLP-1Rs) before feeding, when GLP-1 levels rise in anticipation, reduces food intake, suggesting an appetite-stimulating role for GLP-1 (Vahl et al., 2010). Notably, this effect was reported under highly restricted feeding schedules, raising questions about its relevance in naturalistic contexts (Williams, 2010).

During motivational phases (Figures 1A,B: MOTIVATION), ghrelin enhances reward-seeking behaviors, such as nose pokes for high-fat food pellets, independent of caloric need (Sun et al., 2004; Perello et al., 2010). In rodents, blocking ghrelin receptors abolishes these behaviors (Egecioglu et al., 2010), suggesting a role in sustaining motivational drive. Conversely, GLP-1R activation reduces the motivation to consume palatable foods (Dickson et al., 2012; Howell et al., 2019), underscoring its action as a satiety sensor (Wang et al., 2015). These mechanisms help organisms prioritize high-energy rewards when resources are abundant.

During consumption (Figures 1A,B: CONSUMPTION), ghrelin levels drop in response to caloric intake (Tschöp et al., 2000; Callahan et al., 2004), while satiety peptides such as GLP-1, cholecystokinin (CCK), and peptide YY rise to signal fullness (Murphy and Bloom, 2006). However, elevated ghrelin levels (Monteleone et al., 2012) and diminished CCK responses (Monteleone et al., 2013) during hedonic feeding can override homeostatic regulation, reinforcing consumption even in the absence of need.

Satiation, or the process of learning from hedonic pleasures of reward consumption, parallels associative and reinforcement learning strategies (Figures 1A,B: SATIATION). Although traditionally defined as reduced hunger, in reward literature, satiation more broadly refers to the integration of post-consummatory signals that inform future behavior, including food, social (Kohls et al., 2012), or musical rewards (Witek and Vuust, 2016). This expanded interpretation aligns with findings that gut-derived hormones and peripheral circuits shape learning following reward. For example, GLP-1R agonists restore impaired associative learning in humans (Hanssen et al., 2023) and reduce excitatory drive onto ventral tegmental area (VTA) dopamine neurons projecting to the nucleus accumbens (NAc; Wang et al., 2015). Similarly, optogenetic stimulation of gut-innervating vagal neurons promotes operant self-stimulation, real-time place preference, and flavor conditioning (Han et al., 2018), highlighting gut-brain contributions to reward learning.

While these mechanisms characterize systemic gut-brain communication, peripheral circuitry provides a complementary and fast synaptic pathway (Figures 2A–C). For instance, CCK-expressing EECs mediate flavor preferences through vagal afferents, while serotonin-expressing EECs drive taste aversion via spinal afferents (Bai et al., 2022; Buchanan et al., 2022), rapidly protecting organisms from ingesting harmful substances. Activation of rodent vagal neurons by nutrient-rich foods triggers mesolimbic dopamine release, reinforcing adaptive feeding (Han et al., 2018; Kim et al., 2023; McDougle et al., 2024). This gut-brain communication is also observed in zebrafish (Ye et al., 2020; Isabella et al., 2021) and flies (Kim et al., 2021; Min et al., 2021; Gao et al., 2024), highlighting conserved mechanisms to distinguish beneficial from toxic foods.

In the brain (Figure 2D), anticipation and motivation are primarily driven by the arcuate nucleus of the hypothalamus (ARC), a target of gut hormones, which receives input from the nucleus tractus solitarius (NTS) and modulates mesolimbic and mesocortical reward circuits (Rossi and Stuber, 2018). For instance, ghrelin acts on ARC neuropeptide Y neurons to release orexigenic peptides and promote feeding (Nakazato et al., 2001; Cowley et al., 2003). During consumption, hedonic value is encoded by endogenous opioids acting at “hotspots” within the NAc, orbitofrontal cortex, insular cortex, and interconnected regions of the hypothalamus, VTA, and amygdala (DiFeliceantonio et al., 2012; Morales and Berridge, 2020). GLP-1R agonists suppress intake of palatable food via receptors in the NTS and VTA, reducing synaptic strength onto NAc-projecting VTA dopamine neurons (Alhadeff and Grill, 2014; Wang et al., 2015), whereas blocking GLP-1 signaling promotes consumption of calorie-dense foods (Alhadeff et al., 2012, 2017). Satiation integrates sensory, emotional, and cognitive inputs across distributed circuits to give rise to learning via reward prediction errors (RPEs; Lokshina et al., 2021). While primarily encoded by mesolimbic dopamine, nascent evidence suggests RPEs exist across various regions. For example, amygdalar activity heightens when novel foods are paired with delayed gastrointestinal malaise, but fade in the absence of unexpected consequences, illustrating the role of postingestive feedback in learned aversions (Zimmerman et al., 2025).

Interestingly, ghrelin and GLP-1 influence non-food rewards as well. Ghrelin predicts gambling persistence in humans (Sztainert et al., 2018) and enhances alcohol’s rewarding effects in mice (Jerlhag et al., 2009). Conversely, GLP-1R activation in rodent VTA decreases the motivation to consume cocaine (Schmidt et al., 2016), alcohol (Shirazi et al., 2013), and nicotine (Tuesta et al., 2017), suggesting a conserved role for modulating non-food rewards and holding clinical therapeutic potential (Jerlhag, 2023).

Together these findings demonstrate how gut-derived signals shape central reward circuits by integrating immediate neural responses with sustained hormonal effects to balance hedonic pursuits with survival needs.

### 2.2 Cardiac signals in reward processing

Cardiac interoceptive signals support survival by influencing both homeostatic and hedonic choices (Figure 1C). While gut signals primarily influence natural rewards like food, cardiac fluctuations inform choices involving uncertainty, effort, or risk. These adaptive behaviors include foraging, exploration, or competition, as well as modern analogs like gambling, which engage similar neurophysiological mechanisms (Kimura, 2019; Kimura et al., 2023).

During reward anticipation (Figures 1A,C: ANTICIPATION), humans show parasympathetic-induced heart rate deceleration that orients attention towards ecologically relevant stimuli (Graham and Clifton, 1966), enhances cognitive-motor efficiency to support rapid action (Alam et al., 2023), and emerges before placing gambling bets (Sayão et al., 2021). Mediated by the central autonomic network, this deceleration primes the body for focused effort, much like an animal preparing to evade a predator or initiate a chase (Löw et al., 2008; Panitz et al., 2013), underscoring its role in reward anticipation.

As anticipation shifts to motivated behaviors (Figures 1A,C: MOTIVATION), dopaminergic activity triggers the sympathetic nervous system, shortening the pre-ejection period (PEP) in humans (Ahles et al., 2017). PEP is the interval between left-ventricular depolarization to blood ejection into the aorta and occurs following sympathetic stimulation of the heart via beta_1_-adrenoreceptor activation (Lanfranchi and Somers, 2010). Compared to healthy adults, individuals at risk for neuropsychiatric disorders exhibit exaggerated heart rate changes during alcohol use, gambling, or anhedonia, indicating sympathetic activation and a shortened PEP (Richter and Gendolla, 2009; Brenner and Beauchaine, 2011; Ahles et al., 2017; Silvia et al., 2020). These cardiac signals optimize energy expenditure through shared autonomic and central regulatory circuits (Critchley and Harrison, 2013), balancing between exploring new opportunities and exploiting known resources.

Cardiac signals also shape consumption and learning (Figures 1A,C: CONSUMPTION and SATIATION). For example, negative feedback, such as losing money, triggers a parasympathetic response, while positive feedback activates sympathetic pathways, reinforcing the reward experience (Dekkers et al., 2015; Kastner et al., 2017; Figures 2A,B). Causal evidence from optogenetic studies further supports this link, showing that increasing heart rate in mice induces anxiety-like behaviors in anxiety-provoking contexts (Hsueh et al., 2023). These cardiovascular signals that heighten stress and anxiety modulate decisions by shaping responses to future internal and external cues. Future studies should aim to advance from correlational findings to developing causal relationships between cardiac function and reward processing phases.

The heart-brain axis reflects evolutionary refinements aimed at maintaining cardiovascular stability and facilitating rapid adaptive responses. Parasympathetic vagal neurons (Figure 2A) and sympathetic spinal neurons (Figure 2B) mediate heart-brain communication, allowing the heart to relay sensory information about blood flow and chemical composition to the brain (Prescott and Liberles, 2022; Rajendran et al., 2024). Baroreceptors in the aortic arch, linked to vagal afferents, detect blood pressure changes (Figure 2C) and signal the brain to modulate vagal efferent neurons controlling heart rate, such as those innervating the sinoatrial node, the heart’s pacemaker (Capilupi et al., 2020). Spinal afferents trigger reflexes that elicit sympathetic activity and dopaminergic responses, supporting fight-or-flight behaviors, crucial for survival in dynamic environments (Malliani and Montano, 2002). Like the gut, peripheral afferents from the heart relay to brainstem regions, such as the NTS and parabrachial nucleus (Scott et al., 2025; Figure 2D). These cardiac signals are tightly regulated by the brain’s reward processing centers such as the VTA, amygdala, and ventral striatum (Camara et al., 2009; Lewis et al., 2021; Weinstein, 2023). This neural innervation of the heart is conserved across diverse organisms, including hermit crabs (Yazawa and Kuwasawa, 1994), flies (Dulcis and Levine, 2003), and zebrafish (Stoyek et al., 2015), underscoring its foundational role in cardiovascular regulation and adaptive behaviors.

## 3 Bioenergetic explanations for the body’s role in reward processing

### 3.1 Integration of homeostatic and hedonic mechanisms

The brain’s primary role is to maintain physiological balance by driving behaviors that restore homeostasis. This section examines how physiological signals drive reward processing and motivation, emphasizing the body’s role in adaptive behaviors for survival. Evolution has shaped the brain to prioritize rewarding stimuli that align with bodily needs. Discomfort from hunger, thirst, or social isolation motivates behaviors that restore balance. This process, known as positive alliesthesia, describes how stimuli become more rewarding when they meet homeostatic demands (Cabanac, 1971).

These adaptive mechanisms promote survival by supporting goal-directed responses to internal disruptions. Beyond food and water, social interactions maintain homeostasis, fulfilling emotional and psychological needs, contributing to “social homeostasis” across species (Matthews and Tye, 2019). In this way, these rewards similarly engage homeostatic and hedonic reward pathways to support survival and well-being (Rossi and Stuber, 2018; Matthews and Tye, 2019; Grove et al., 2022; Wee et al., 2024).

### 3.2 Motivational intensity theory

The classical motivational intensity theory posits that an organism’s energy expenditure is proportional to the difficulty of obtaining a reward, up until the required effort surpasses the perceived value (Richter and Gendolla, 2009; Richter et al., 2016). This framework highlights how gut-derived and cardiac signals modulate energy allocation during reward pursuit.

Gut signals influence motivational intensity by integrating physiological readiness with behavioral drive. Ghrelin enhances the perceived value of energy-rich foods and promotes effortful food-seeking (Perello et al., 2010), while GLP-1 dampens motivation for palatable (Dickson et al., 2012) and non-food rewards (Egecioglu et al., 2013a, 2013b). Beyond reward signaling, ghrelin-mediated anticipatory digestive processes represent a metabolic investment, aligning energetic costs of digestive readiness with expected intake (Masuda et al., 2000; Secor, 2009). Insufficient preparation may impair digestion or promote microbial overgrowth, underscoring ghrelin’s role as a metabolic “bet,” balancing effort with internal needs.

Cardiac responses similarly reflect the body’s energy expenditure during different phases of reward processing. Parasympathetic activity conserves energy during anticipation, preparing the body for action (Lovallo and Sollers, 2007). It acts as a real-time biomarker for prioritizing responses to environmental stimuli (Richter et al., 2016), including food (Zebunke et al., 2011), social (Zupan et al., 2016), and sexual cues (Creswell et al., 2013) across species, including pigs, dogs, and humans. During reward-seeking, sympathetic activity increases with task difficulty, but decreases when effort outweighs the reward’s value (Richter, 2010), selectively mobilizing energy.

Evolutionarily, these integrated mechanisms ensure strategic allocation of energy toward high-value rewards, optimizing resource acquisition and adaptability.

### 3.3 Predictive interoception coding

Predictive interoception coding provides a framework for how the brain anticipates and integrates internal bodily signals to maintain homeostasis and guide reward-related behaviors. The brain’s ability to generate internal expectations has evolved from simple reflexes to complex predictive models (Pezzulo et al., 2021). For example, false heart rate feedback can create interoceptive illusions, making participants perceive greater effort during exercise when they believe their heart rate is elevated (Iodice et al., 2019), illustrating the brain’s reliance on predicted internal states to calibrate effort. Beyond homeostasis, predictive interoception also shapes emotional and reward-based decisions (Seth, 2013). In a gambling task, participants with greater anticipatory awareness of emotional states made faster, more advantageous financial decisions (Marshall et al., 2019).

Understanding the interaction between reward circuits and the peripheral nervous system reflects how predictive mechanisms have adapted to regulate physiology, motivation, and reward seeking. This integration highlights the brain’s role in optimizing responses to internal and external challenges, enhancing survival.

## 4 Therapeutic implications of interoception

Neural processing of peripheral organ signals regulates stress, enhances resilience, and holds promise for neuropsychiatric treatment (Benton et al., 2021; Natterson-Horowitz et al., 2023). In humans, gut-derived signals influence emotional states and reward processing. For example, intragastric fat infusion attenuates experimentally induced sadness and dampens activity in emotion-related brain regions (Van Oudenhove et al., 2011), while striatal dopamine release during pleasurable meals predicts subjective pleasure ratings (Small et al., 2003). These psychophysical links between the gut and emotional experience underscore the promise of interoceptive therapeutics.

Originally developed for diabetes and weight management, GLP-1R agonists are now being explored for reducing cravings in addiction and mitigating symptoms of psychotic and neurocognitive disorders (Klausen et al., 2022; Leggio et al., 2023; Xie et al., 2025). Similarly, heart rate variability, a physiological marker of emotional regulation and reward sensitivity, has emerged as a therapeutic target, showing efficacy in reducing substance (Eddie et al., 2014), alcohol (Penzlin et al., 2015), and food cravings (Meule et al., 2012).

Looking ahead, interoceptive therapeutics, including trainings to enhance bodily awareness and advanced vagus nerve stimulation (VNS), hold promise for improving emotional resilience and mental health (Khalsa et al., 2017; Kim et al., 2024; Schuman-Olivier et al., 2024). Although current VNS techniques show efficacy in treating conditions like depression and anxiety, improving specificity remains a challenge. Technologies like optogenetics and targeted gene delivery aim to define cell-type-specific neuromodulation, minimizing off-target effects and enhancing efficacy (Bansal et al., 2023). Collectively, brain–body interventions hold immense potential for the treatment of various conditions, including depression, autism, and anxiety disorders (Paulus and Stein, 2010; DuBois et al., 2016; Rogers et al., 2016).

## 5 Conclusion

This mini-review provides an adaptive framework for understanding brain–body communication in reward processing. While focused on select interoceptive signals, other sensory inputs (e.g., gut microbiota) and organs (e.g., pancreas) also shape reward behaviors (Davis et al., 2010; Kim et al., 2023). A truly holistic view requires acknowledging multi-organ interactions, such as gut-heart-brain communication. For example, duodenal glucose infusions can lower blood pressure in healthy adults (O’Donovan et al., 2002), while gastric distension raises arterial pressure via sympathetic activation to offset digestive-related blood redistribution (Rossi et al., 1998). Such visceral signals likely modulate reward circuits by influencing physiological states. Simultaneous investigation of these systems allows for the discovery of both direct and emergent properties that govern brain–body communication, underscoring the need to integrate such complexities into a holistic view of reward processing and its role in survival and well-being.
